# Lycopodine-Type Alkaloids from *Lycopodium japonicum*

**DOI:** 10.1007/s13659-014-0027-1

**Published:** 2014-07-17

**Authors:** Juan He, Xing-De Wu, Fei Liu, Yu-Cheng Liu, Li-Yan Peng, Yu Zhao, Xiao Cheng, Huai-Rong Luo, Qin-Shi Zhao

**Affiliations:** State Key Laboratory of Phytochemistry and Plant Resources in West China, Kunming Institute of Botany, Chinese Academy of Sciences, Kunming, 650201 China

**Keywords:** *Lycopodium japonicum*, Lycopodine-type alkaloids, 4*α*-Hydroxyanhydrolycodoline, 4*α*,6*α*-Dihydroxyanhydrolycodoline, 6-*epi*-8*β*-Acetoxylycoclavine, Lycoposerramine G nitrate

## Abstract

Three new lycopodine-type alkaloids, 4*α*-hydroxyanhydrolycodoline (**1**), 4*α*,6*α*-dihydroxyanhydrolycodoline (**2**), and 6-*epi*-8*β*-acetoxylycoclavine (**3**), and an artifact, lycoposerramine G nitrate (**4**), along with seventeen related known compounds, were isolated from the club moss *Lycopodiumjaponicum* Thunb. ex Murray (Lycopodiaceae). Their structures were elucidated by extensive spectroscopic methods as well as X-ray analysis. Compounds **1**–**4** were evaluated for their acetylcholine esterase inhibitory activity.

**Electronic supplementary material:**

The online version of this article (doi:10.1007/s13659-014-0027-1) contains supplementary material, which is available to authorized users.

## Introduction

*Lycopodium* alkaloids have attracted great interests of phytochemists and synthetic chemists for a long time due to their complicated structures as well as potent biological activities [1–5]. Till now, more than 300 *Lycopodium* alkaloids have been obtained [6, 7], which were classified into four structural types by chemist Ayer [8], namely, lycopodine-type, lycodine-type, fawcettimine-type, and miscellaneous-type.

**Fig. 1:**
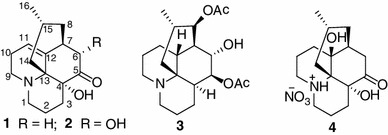
Compounds **1**–**4** isolated from *L.japonicum*

*Lycopodiumjaponicum* Thunb. ex Murray, abundant in Guangdong, Guangxi, Yunnan, and Guizhou provinces of China, was historically used as a traditional folk medicine for the treatment of contusion, strains, and myasthenia. Its chemical constituents have been widely investigated and a large number of compounds have been isolated [9–14]. Our previous study on this plant reported a novel *Lycopodium* alkaloid, lycojapodine A [15, 16]. A continuous study on the same plant led to the isolation of three new lycopodine-type alkaloids, 4*α*-hydroxyanhydrolycodoline (**1**), 4*α*,6*α*-dihydroxyanhydrolycodoline (**2**), and 6-*epi*-8*β*-acetoxylycoclavine (**3**), and an artifact, lycoposerramine G nitrate (**4**) (Fig. 1), together with seventeen related known compounds. Compounds **1**–**4** were tested for their acetylcholine esterase (AChE) inhibitory activity, yet no positive results were observed. Herein, we report the isolation and structural elucidation of these compounds.

## Results and Discussion

The crude base extract of *L. japonicum* was separated by normal-phase silica gel, RP-18 silica gel, and Sephadex LH-20 chromatography to afford twenty-one lycopodine-type alkaloids, seventeen of which were known ones. The structures of known compounds, compared with literature data, were identified as lycopodine [17], clavolonine [17], alkaloid L-23 [17], lucidioline [18], alkaloid L-20 [18], lycoposerramine M [18], lycoposerramine G [18], lycoposerramine K [18], lycoposerramine F [18], anhydrolycodoline [19], lycoclavine [20], serratezomine C [21], huperzine E [22], 12-epilycodoline-*N*-oxide [23], diphaladine A [24], 12-deoxyhuperzine O [25], and 8*β*-acetoxy-11*α*-hydroxylycopodine [26].

**Fig. 2:**
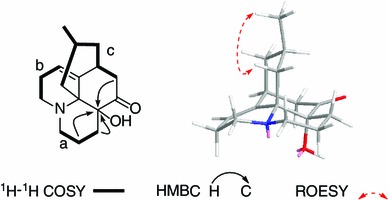
Key 2D NMR correlations of compound **1**

Compound **1** was obtained as a colorless crystal. Its molecular formula, C_16_H_23_NO_2_, was established by HRESIMS ([M+H]^+^ at *m*/*z* 262.1806). The ^1^H NMR (Table 1) spectrum of **1** displayed one methyl (*δ*_H_ 0.84, d, *J* = 6.2 Hz) and one olefinic proton (*δ*_H_ 5.65, d, *J* = 5.1 Hz). The ^13^C NMR and DEPT spectra of **1** (Table 2) exhibited 16 carbon resonances due to four quaternary carbons (one oxygenated at *δ*_C_ 75.2, one olefinic at *δ*_C_ 139.9, and one carbonyl at *δ*_C_ 210.5), three methines (one olefinic at *δ*_C_ 118.8), eight methylenes, and one methyl group at *δ*_C_ 22.1. The ^1^H–^1^H COSY and HSQC data revealed three partial structures: **a** CH_2_CH_2_CH_2_, **b** CH_2_CH_2_CH, and **c** CH_2_CHCH_2_CH(CH_3_)CH_2_ (Fig. 2). Further detailed 2D NMR analysis indicated compound **1** was closely related to anhydrolycodoline [19]. The only difference was that **1** possessed an OH additional group, which was suggested to be connected to C-4 as inferred from the HMBCs of *δ*_H_ 2.25 (1H, *d*, *J* = 15.2 Hz, H-6b), 1.58 (1H, *br. d*, *J* = 12.1 Hz, H-2b), and 1.25 (1H, *m*, H-14b) with C-4.

**Table 1:** ^1^H NMR spectroscopic data for **1**–**4** in CDCl_3_; *J* in Hz and *δ* in ppm

No.	1^a^	2^a^	3^b^	4^a^
1	2.83 (*t*, 13.2)	2.83 (*m*)	3.28 (*td*, 14.0, 3.2)	3.48 (*td*, 16.2, 4.6)
	2.42^c^	2.43^c^	2.49 (*m*)	2.89 (*dd*, 13.0, 4.6)
2	1.89 (*m*)	1.87^c^	1.91 (*m*)	2.22 (*m*)
	1.58 (*br. d*, 12.1)	1.57 (*m*)	1.31 (*m*)	1.49 (*m*)
3	1.87 (*m*)	1.87^c^	1.68^c^	1.89^c^
	1.69 (*m*)	1.69 (*m*)	1.37 (*m*)	1.89^c^
4			2.72 (*m*)	
5			4.84 (*d*, 6.7)	
6	3.12 (*dd*, 15.2, 6.6)	3.86 (*br. s*)	3.77 (*br. s*)	3.26 (*dd*, 16.1, 5.6)
	2.25 (*d*, 15.2)			2.19 (*d*, 16.1)
7	2.76^c^	2.77^c^	2.33 (*d*, 6.5))	2.11 (*br. d*)
8	1.77 (*d*, 13.0)	1.81 (*m*)	4.55 (*dd*, 11.0, 5.2)	1.96 (*m*)
	1.23 (*m*)	1.26 (*m*)		1.32 (*dd*, 13.5, 6.1)
9	2.76^c^	2.77^c^	3.12 (*td*, 12.4, 2.5)	4.31 (*td*, 12.0, 4.6)
	2.56 (*br. s*)	2.58 (*dd*, 10.9, 6.1)	2.47 (*m*)	3.02^*c*^
10	2.42^c^	2.43^c^	1.68^c^	2.46 (*m*)
	1.90 (*m*)	1.97 (*br. d*, 17.1)	1.52 (*m*)	1.32 (*m*)
11	5.65 (*d*, 5.1)	5.79 (*d*, 5.3)	1.88 (*m*)	3.02^c^
			1.43 (*m*)	1.47^c^
12			2.11 (*m*)	
14	2.11 (*dd*, 12.8, 3.5)	2.10 (*dd*, 12.9, 4.0)	2.64 (*dd*, 13.2, 6.5)	1.81^c^
	1.25 (*m*)	1.29 (*m*)	0.97 (*t*, 13.2)	1.81^c^
15	1.66 (*m*)	1.52 (*m*)	2.32 (*m*)	1.47^c^
16	0.84 (3H, *d*, 6.2)	0.83 (3H, *d*, 6.2)	0.87 (3H, *d*, 6.2)	0.81 (3H, d, 6.1)
OAc-5			2.04 (3H, *s*)	
OAc-8			2.00 (3H, *s*)	

^a^Measured on a Bruker DRX-500 MHz

^b^Measured on a Bruker AV-400 MHz

^c^Overlapped

**Table 2:** ^13^C NMR spectroscopic data for **1**–**4** in CDCl_3_; *J* in Hz and *δ* in ppm

No.	1^a^	2^a^	3^b^	4^b^
1	46.5 CH_2_	46.3 CH_2_	47.4 CH_2_	46.8 CH_2_
2	20.0 CH_2_	19.7 CH_2_	19.6 CH_2_	14.4 CH_2_
3	23.9 CH_2_	23.9 CH_2_	23.3 CH_2_	24.3 CH_2_
4	75.2 C	77.0 C	27.3 CH	75.8 C
5	210.5 C	207.3 C	76.4 CH	205.9 C
6	43.3 CH_2_	79.5 CH	69.2 CH	39.8 CH_2_
7	40.4 CH	49.3 CH	45.2 CH	38.5 CH
8	44.0 CH_2_	39.4 CH_2_	79.6 CH	41.0 CH_2_
9	45.1 CH_2_	45.0 CH_2_	46.8 CH_2_	50.3 CH_2_
10	25.6 CH_2_	25.9 CH_2_	36.4 CH_2_	17.8 CH_2_
11	118.8 CH	123.2 CH	25.9 CH_2_	29.2 CH_2_
12	139.9 C	144.6 C	42.6 CH	69.6 C
13	63.5 C	64.2 C	54.4 C	65.9 C
14	34.3 CH_2_	33.7 CH_2_	40.8 CH_2_	35.4 CH_2_
15	24.7 CH	24.9 CH	29.7 CH	24.2 CH
16	22.1 CH_3_	22.8 CH_3_	19.6 CH_3_	22.1 CH_3_
OAc-5			170.6 C	
			21.1 CH_3_	
OAc-8			170.6 C	
			21.1 CH_3_	

^a^Measured on a Bruker DRX-500 MHz

^b^Measured on a Bruker AV-400 MHz

**Fig. 3:**
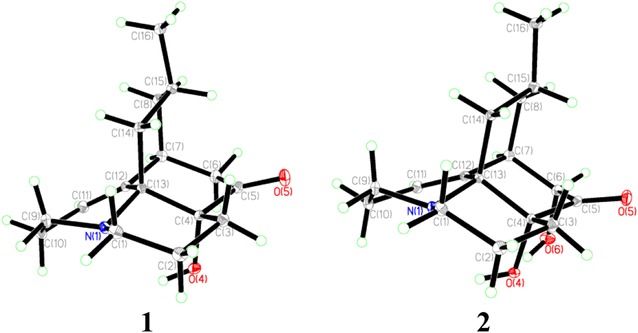
The X-ray structures of compounds **1**–**2**

In the ROESY spectrum of **1**, the correlation of H-14a with Me-16 was observed (Fig. 2). However, due to overlapped signals of H-1b with H-10a and H-9a with H-7, the ROESY spectrum could not provide more sufficient information to elucidate the stereochemistry of **1**. The relative configuration of **1** was established by X-ray analysis (Fig. 3), which validated the *α*-orientition of OH-4, H-7, and Me-16. Therefore, the structure of compound **1** was established as 4*α*-hydroxyanhydrolycodoline.

Compound **2** was isolated a colorless crystal. The HRESIMS displayed an [M+H]^+^ peak at *m*/*z* 278.1748 (corresponding to a molecular formula C_16_H_23_NO_3_), 16 mass unit higher than that of **1**. Comparison of the 1D NMR data (Tables 1 and 2) with those of **1**, compound **2** was readily identified as 6-hydroxy derivative of **1** as deduced from the HMBCs of *δ*_H_ 3.86 (1H, *br. s*, H-6) with *δ*_C_ 39.4 (*t*, C-8), 77.0 (*s*, C-4), and 207.3 (*s*, C-5). The relative configuration of **2** was also established by X-ray analysis, which validated the *α*-orientation of OH-4, OH-6, H-7, and Me-16 (Fig. 3). Thus, the structure of **2** was elucidated as 4*α*,6*α*-dihydroxyanhydrolycodoline.

**Fig. 4:**
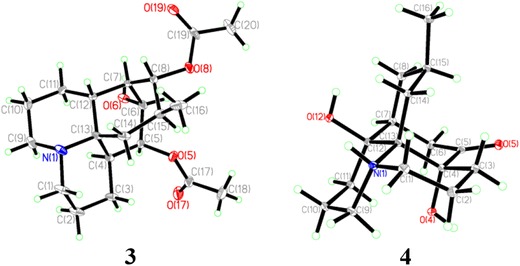
The X-ray structures of compounds **3** and **4**

The molecular formula of compound **3** was determined as C_20_H_31_NO_5_ on the basis of its HRESIMS ([M+H]^+^ at *m*/*z* 366.2270), indicating 6° of unsaturation. IR absorption bands implied the presence of ketone (1738 cm^−1^) and OH (3472 cm^−1^) groups. The ^1^H and ^13^C NMR (Tables 1 and 2) spectra revealed the existence of two OAc groups, seven *sp*^3^ methylenes, seven *sp*^3^ methines (three oxygenated at *δ*_C_ 69.2, 76.4, and 79.6), one *sp*^3^ quaternary carbon, and one methyl group. The above data indicated that **3** had a similar structure to that of lycoclavine [20], except for the existence of an additional OAc group which was located at C-8 according to the HMBCs of *δ*_H_ 2.11 (1H, *m*, H-12), 2.64 (1H, *dd*, *J* = 13.2, 6.5 Hz, H-14a), and 0.87 (3H, *d*, *J* = 6.2 Hz, Me-16) with *δ*_C_ 79.6 (*d*, C-8) as well as *δ*_H_ 4.55 (1H, *dd*, *J* = 11.0, 5.2 Hz, H-8) with *δ*_C_ 170.6. To establish the relative configuration, an X-ray experiment was evolved, which suggested the relative configuration of H-4, H-5, H-6, H-8, H-12, and H-15 to be *α*, *α*, *β*, *α*, *β*, and *β*, respectively (Fig. 4). Thus, the structure of compound **3** was elucidated and named as 6-*epi*-8*β*-acetoxylycoclavine.

Compound **4** had a molecular formula of C_16_H_25_NO_3_, the same as that of lycoposerramine G [18], a known compound also isolated this time. The NMR data and detailed 2D analysis indicated the two compounds had the same planar structure. However, according to the ROESY spectrum, the two compounds also possessed the same relative configuration which indicated **4** should be a salt form of lycoposerramine G. Therefore, a X-ray experiment was implemented that confirmed **4** was lycoposerramine G nitrate (Fig. 4), which was produced during the isolation as verified by the TLC (Al_2_O_3_).

The compounds **1**–**4** were tested for acetylcholine esterase (AChE) inhibitory activity, yet no positive results were observed.

## Experimental Section

### General Experimental Procedures

Melting points were obtained on an WRX-4 micro melting point apparatus (Shanghai Yice Instrument Co., Ltd., Shanghai, China). Optical rotations were measured with JASCO P-1020 digital polarimeter (JASCO, Tokyo, Japan). UV spectra were obtained using a Shimadzu UV-2401A spectrophotometer (Shimadzu, Kyoto, Japan). A Tenor-27 FT infrared spectrophotometer (Bruker Optics, Ettlingen, Germany) was used for scanning IR spectrum using KBr pellets. ESIMS were recorded on an Agilent 6530 Q-Tof spectrometer (Agilent, Palo Alto, CA, USA). HREIMS were measured using a Waters Auto Premier P776 spectrometer (Waters, Milford, MA, USA). 1D and 2D spectra were run on Bruker AV-400 and DRX-500 spectrometers (Bruker Optics, Ettlingen, Germany). Chemical shifts (*δ*) were expressed in ppm with reference to the solvent signals. Column chromatography (CC) was performed on Silica gel (200–300 mesh, Qingdao Marine Chemical Ltd., Qingdao, China), RP-18 gel (20–45 µm, Fuji Silysia Chemical Ltd., Japan), and Sephadex LH-20 (GE healthcare Bio-sciences AB, Sala, Sweden). Semipreparative HPLC was performed on an Agilent 1100 liquid chromatograph (Agilent, Palo Alto, CA, USA). Liquid chromatograph was equipped with a UV detector (190–400 nm) and a Zorbax SB-C_18_ (9.4 mm × 25 cm column, particle size 5 μm, 1–3 mL/min). Fractions were monitored by Thin-layer chromatography (TLC) (GF_254_, Qingdao Haiyang Chemical Co. Ltd., Qingdao, China), and spots were visualized by heating silica gel plates sprayed with 10 % H_2_SO_4_ in EtOH or by Dragendorff’s reagent.

### Plant Material

The whole plants of *L. japonicum* were collected in Simao of Yunnan Province, People’s Republic of China, in August 2006. The sample was identified by Prof. Xiao Cheng at Kunming Institute of Botany, Chinese Academy of Sciences (vocher no. 2006-8-17).

### Extraction and Isolation

Air-dried, powdered sample (50 kg) of *L.japonicum* was dealt as the method reported before to obtain an base extract (67 g) [15]. This extract was subjected to a silica gel column chromatography (CC) with a gradient elution system of petroleum ether–actone (1:0–0:1) to give 7 fractions (I–VI). Fraction I (7 g) was chromatographed over several silica gel CC eluted with petroleum ether–EtOAc (9:1–1:1) to give three sub-fractions, I-a, I-b, and I-c. I-b was purified by (CHCl_3_–MeOH, 1:1) to yield **1** (8 mg), 12-deoxyhuperzine O (14 mg), and huperzine E (7 mg). I-c was repeatedly purified by silica gel CC (petroleum ether–acetone) to afford anhydrolycodoline (40 mg) and lycoposerramine K (27 mg). Faction II (14 g) was eluted by silica gel CC (petroleum ether–acetone) to afford four sub-fractions, II-a, II-b, II-c, and II-d. II-a repeatedly purified by silica gel CC (petroleum ether–acetone, 8:2) to obtained **2** (7 mg) and lycopodine (14 mg). Alkaloid L-20 was obtained from II-b by recrystallisation. After repeatedly purified by silica gel CC (CHCl_3_–acetone) and Sephadex LH-20 (CHCl_3_–MeOH, 1:1), II-c give compound clavolonine (11 mg). Faction III (6 g) was purified by silica gel CC (CHCl_3_–MeOH, 9.8:0.2), Sephadex LH-20 CC (CHCl_3_–MeOH, 1:1), and semipreparative HPLC (MeOH–H_2_O, 85:15) to afford **3** (10 mg) and 8*β*-acetoxy-11*α*-hydroxylycopodine (3 mg). Fraction IV (9 g) was subjected to silica gel CC (CHCl_3_–MeOH, 9.5:0.5) to give three sub-fractions: IV-a, IV-b, and IV-c. Fraction IV-a was purified by recrystallization and silica gel CC (CHCl_3_–MeOH, 9.8:0.2) to afford lycoposerramine M (16 mg), lycoclavine (5 mg), and 12-epilycodoline-*N*-oxide (11 mg). IV-b was subjected to silica gel CC (CHCl_3_–MeOH, 9:1) to yield alkaloid L-23 (21 mg) and lycodoline (54 mg). Fraction IV-c was subjected to silica gel CC (CHCl_3_–MeOH, 9.5:0.5) and then purified by recrystallization to afford **4** (9 mg) and serratezomine C (8 mg). Fraction-V (11 g) was subjected to silica gel CC (CHCl_3_–MeOH, 9:1) and further purified by RP-18 CC (MeOH–H_2_O, 4:6–7:3) to yield lucidioline (25 mg), lycoposerramine G (11 mg), and lycoposerramine F (10 mg). Fraction-VI (8 g) was purified by RP-18 CC (MeOH–H_2_O, 5:5) to afford diphaladine A (13 mg).

### Acetylcholinesterase Inhibition

Acetylcholinesterase (AChE) inhibitory activity of the compounds **1**–**4** isolated was assayed by the spectrophotometric method developed by Ellman et al. [27] with slightly modification. *S*-Acetylthiocholine iodide, *S*-butyrylthiocholine iodide, 5,5′-dithio-bis-(2-nitrobenzoic) acid (DTNB, Ellman’s reagent), acetylcholinesterase derived from human erythrocytes were purchased from Sigma Chemical. Compounds were dissolved in DMSO. The reaction mixture (totally 200 μL) containing phosphate buffer (pH 8.0), test compound (50 μM), and acetyl cholinesterase (0.02 U/mL), was incubated for 20 min (30 °C). Then, the reaction was initiated by the addition of 40 μL of solution containing DTNB (0.625 mM) and acetylthiocholine iodide (0.625 mM) for AChE inhibitory activity assay, respectively. The hydrolysis of acetylthiocholine was monitored at 405 nm every 30 s for 1 h. Tacrine was used as positive control with final concentration of 0.333 μM. All the reactions were performed in triplicate. The percentage inhibition was calculated as follows: % inhibition = (E − S)/E × 100 (E is the activity of the enzyme without test compound and S is the activity of enzyme with test compounds).

### 4*α*-Hydroxyanhydrolycodoline (**1**)

Colorless crystal (MeOH); mp 129–130 °C; $[\alpha ]_{\text{D}}^{ 2 6} - \; 1 6 3. 50 \, \left( {c\;0.0 1,{\text{ CHCl}}_{ 3} } \right)$; UV (MeOH) *λ*_max_ (log *ε*) 202 (2.85) nm; IR (KBr) *ν*_max_ 3362, 2919, 1711 cm^−1^; ^1^H and ^13^C NMR data, see Tables 1 and 2; positive HRESIMS *m/z* 262.1806 (calcd for C_16_H_24_NO_2_ [M+H]^+^, 262.1807).

### Crystal Data for 4*α*-Hydroxyanhydrolycodoline (**1**)

C_16_H_23_NO_2_, *M* = 261.35; orthorhomic, space group *P*2_1_2_1_2_1_; *a* = 7.4471 (7) Å, *b* = 9.7363 (9) Å, *c* = 9.2184 (9) Å, *α* = 90.00, *β* = 90.6970, *γ* = 90.00, *V* = 668.35 (11) Å^3^, *Z* = 2, *μ*(MoKα) = 0.085 mm^−1^, crystal dimensions 0.14 × 0.23 × 0.45 mm was used for measurement on a Bruker APEX DUO diffractometer using graphitemonochromated MoKα radiation. The total number of reflections measured was 7187, of which 3461, were observed, *I* > 2σ(*I*). Final indices: *R*_1_ = 0.0326, w*R*_2_ = 0.0912. Crystallographic data for the structure of **1** have been deposited in the Cambridge Crystallographic Data Centre (deposition number CCDC 870095). Copies of the data can be obtained free of charge from the CCDC via www.ccdc.cam.ac.uk.

### 4*α*,6*α*-Dihydroxyanhydrolycodoline (**2**)

Colorless crystal (MeOH); mp 157–158 °C; $[\alpha ]_{\text{D}}^{ 2 6} - \; 9 1. 2 7 { }\left( {c\;0.0 1,{\text{ CHCl}}_{ 3} } \right)$; UV (MeOH) *λ*_max_ (log *ε*) 203 (3.05), 264 (3.09) nm; IR (KBr) *ν*_max_ 3463, 3431, 2921, 1722 cm^−1^; ^1^H and ^13^C NMR data, see Tables 1 and 2; positive HRESIMS *m/z* 278.1748 (calcd for C_16_H_24_NO_3_ [M+H]^+^, 278.1756).

### Crystal Data for 4*α*,6*α*-Dihydroxyanhydrolycodoline (**2**)

C_16_H_23_NO_3_, *M* = 277.35; orthorhomic, space group *P*2_1_2_1_2_1_; *a* = 7.7550 (8) Å, *b* = 8.8037 (9) Å, *c* = 10.1722 (9) Å, *α* = 90.00, *β* = 98.2380, *γ* = 90.00, *V* = 687.32 (12) Å^3^, *Z* = 2, *μ*(MoKα) = 0.092 mm^−1^, crystal dimensions 0.33 × 0.40 × 0.40 mm was used for measurement on a Bruker APEX APEX DUO diffractometer using graphitemonochromated MoKα radiation. The total number of reflections measured was 7457, of which 3525, were observed, *I* > 2σ(*I*). Final indices: *R*_1_ = 0.0335, w*R*_2_ = 0.0865. Crystallographic data for the structure of **2** have been deposited in the Cambridge Crystallographic Data Centre (deposition number CCDC 870093). Copies of the data can be obtained free of charge from the CCDC via www.ccdc.cam.ac.uk.

### 6-*epi*-8*β*-Acetoxylycoclavine (**3**)

Colorless crystal (MeOH); mp 170–171 °C; $[\alpha ]_{\text{D}}^{ 2 6} \; + \; 40. 6 3 { }\left( {c\;0.0 1,{\text{ CHCl}}_{ 3} } \right)$; UV (MeOH) *λ*_max_ (log *ε*) 203 (2.89) nm; IR (KBr) *ν*_max_ 3472, 2937, 1738, 1236, 1030 cm^−1^; ^1^H and ^13^C NMR data, see Tables 1 and 2; positive HRESIMS *m/z* 366.2270 (calcd for C_20_H_32_NO_5_ [M+H]^+^, 366.2280).

### Crystal Data for 6-*epi*-8*β*-acetoxylycoclavine (**3**)

C_20_H_33_NO_6_ (C_20_H_31_NO_5_+H_2_O), *M* = 383.47; orthorhomic, space group *P*2_1_2_1_2_1_; *a* = 9.364 (3) Å, *b* = 12.755 (4) Å, *c* = 9.528 (3) Å, *α* = 90.00, *β* = 119.105, *γ* = 90.00, *V* = 994.3 (5) Å^3^, *Z* = 2, *μ*(MoKα) = 0.094 mm^−1^, crystal dimensions 0.05 × 0.05 × 0.60 mm was used for measurement on a Bruker APEX APEX DUO diffractometer using graphitemonochromated MoKα radiation. The total number of reflections measured was 13705, of which 3167, were observed, *I* > 2σ(*I*). Final indices: *R*_1_ = 0.0580, w*R*_2_ = 0.1206. Crystallographic data for the structure of **3** have been deposited in the Cambridge Crystallographic Data Centre (deposition number CCDC 970097). Copies of the data can be obtained free of charge from the CCDC via www.ccdc.cam.ac.uk.

### Lycoposerramine G Nitrate (**4**)

Colorless crystal (MeOH); $[\alpha ]_{\text{D}}^{ 2 6} - \; 4 7. 2 1 { }\left( {c\;0.0 1,{\text{ CHCl}}_{ 3} } \right)$; UV (MeOH) *λ*_max_ (log *ε*) 202 (1.93) nm; IR (KBr) *ν*_max_ 3421, 2924, 1720, 1439 cm^−1^; ^1^H and ^13^C NMR see Tables 1 and 2; positive HRESIMS *m/z* 280.1913 (calcd for C_16_H_26_NO_3_ [M+H]^+^, 280.1912).

### Crystal Data for Lycoposerramine G Nitrate (**4**)

C_16_H_28_N_2_O_7_ (C_16_H_26_NO_3_^+^+ NO_3_^−^+ H_2_O), *M* = 360.40; orthorhomic, space group *P*2_1_2_1_2_1_; *a* = 8.5470 (8) Å, *b* = 8.8731 (8) Å, *c* = 21.963 (2) Å, *α* = 90.00, *β* = 90.00, *γ* = 90.00, *V* = 1665.6 (3) Å^3^, *Z* = 4, *μ*(MoKα) = 0.112 mm^−1^, crystal dimensions 0.30 × 0.33 × 0.90 mm was used for measurement on a Bruker APEX APEX DUO diffractometer using graphitemonochromated MoKα radiation. The total number of reflections measured was 17689, of which 4580, were observed, *I* > 2σ(*I*). Final indices: *R*_1_ = 0.0277, w*R*_2_ = 0.0756. Crystallographic data for the structure of **4** have been deposited in the Cambridge Crystallographic Data Centre (deposition number CCDC 1001519). Copies of the data can be obtained free of charge from the CCDC via www.ccdc.cam.ac.uk.

### Crystal Data for Lycoposerramine G

C_32_H_50_N_2_O_6_ (2 × C_16_H_25_NO_3_), *M* = 558.74; orthorhomic, space group *P*2_1_2_1_2_1_; *a* = 8.2885 (7) Å, *b* = 15.8779 (13) Å, *c* = 21.1225 (17) Å, *α* = 90.00, *β* = 90.00, *γ* = 90.00, *V* = 2813.3 (4) Å^3^, *Z* = 4, *μ*(MoKα) = 0.090 mm^−1^, crystal dimensions 0.29 × 0.43 × 0.43 mm was used for measurement on a Bruker APEX APEX DUO diffractometer using graphitemonochromated MoKα radiation. The total number of reflections measured was 30120, of which 7570, were observed, *I* > 2σ(*I*). Final indices: *R*_1_ = 0.0320, w*R*_2_ = 0.0837. Crystallographic data for the structure of lycoposerramine G have been deposited in the Cambridge Crystallographic Data Centre (deposition number CCDC 1001520). Copies of the data can be obtained free of charge from the CCDC via www.ccdc.cam.ac.uk.

## Electronic supplementary material

Below is the link to the electronic supplementary material.

Supplementary material 1 (DOC 716 kb)
