# Supplementary material for: Lycopodine-Type Alkaloids from Lycopodium japonicum
Source: Nat Prod Bioprospect. 2014 Jul 17;4(4):213–9. doi: 10.1007/s13659-014-0027-1 (PMC4111880; doi:10.1007/s13659-014-0027-1)

Supporting Information for

**Lycopodine-type Alkaloids from *Lycopodium japonicum***

Juan He, Xing-De Wu, Fei Liu, Yu-Cheng Liu, Li-Yan Peng, Yu Zhao, Xiao Cheng, Huai-Rong Luo, Qin-Shi Zhao*

**Affliliation**

State Key Laboratory of Phytochemistry and Plant Resources in West China, Kunming Institute of Botany, Chinese Academy of Science, Kunming 650201, China

**Correspondences**

Prof Qin-Shi Zhao, State Key Laboratory of Phytochemistry and Plant Resources in West China, 132 Lanhei road, Kunming 650201, P. R. China. E-mail: [qinshizhao@mail.kib.ac.cn](mailto:qinshizhao@mail.kib.ac.cn). Phone: +8687165223058 Fax: +8687165215783.

Contents of Supporting Information

| **Contents** | **page** |
| --- | --- |
| Figure 1S. X-ray structures for **lycoposerramine G** | 3 |
| Figure S1-S6. NMR and MS spectra for **1** in CDCl_3_ | 4-6 |
| Figure S7-S12. NMR and MS spectra for **2** in CDCl_3_ | 7-9 |
| Figure S13-S18. NMR and MS spectra for **3** in CDCl_3_ | 10-12 |
| Figure S19-S25. NMR spectra for **4** in CDCl_3_ | 13-16 |

Figure 1S. X-ray structures for **lycoposerramine G**.

**
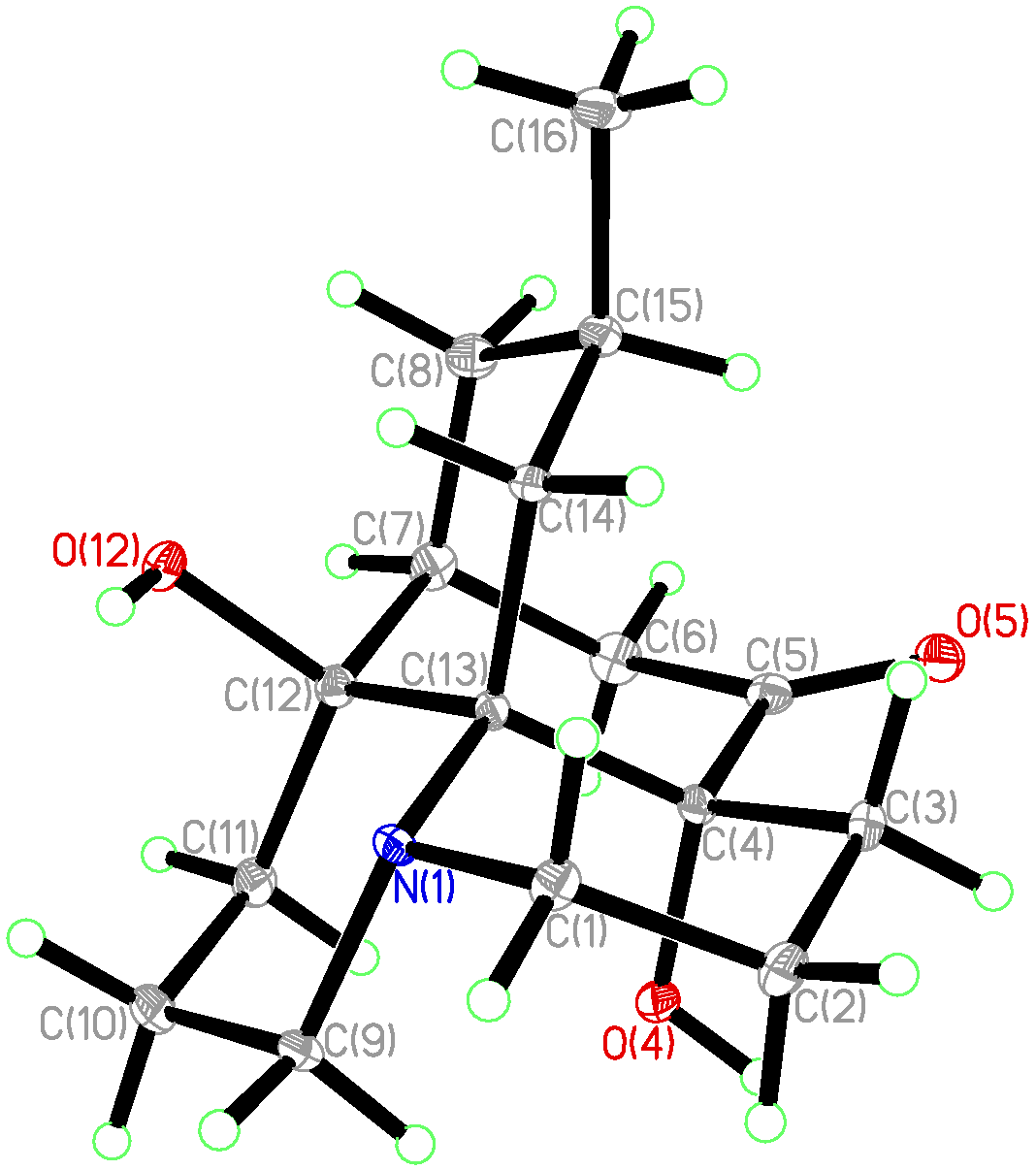
**

Figure S1. ^1^H of compound **1** in CDCl_3_.


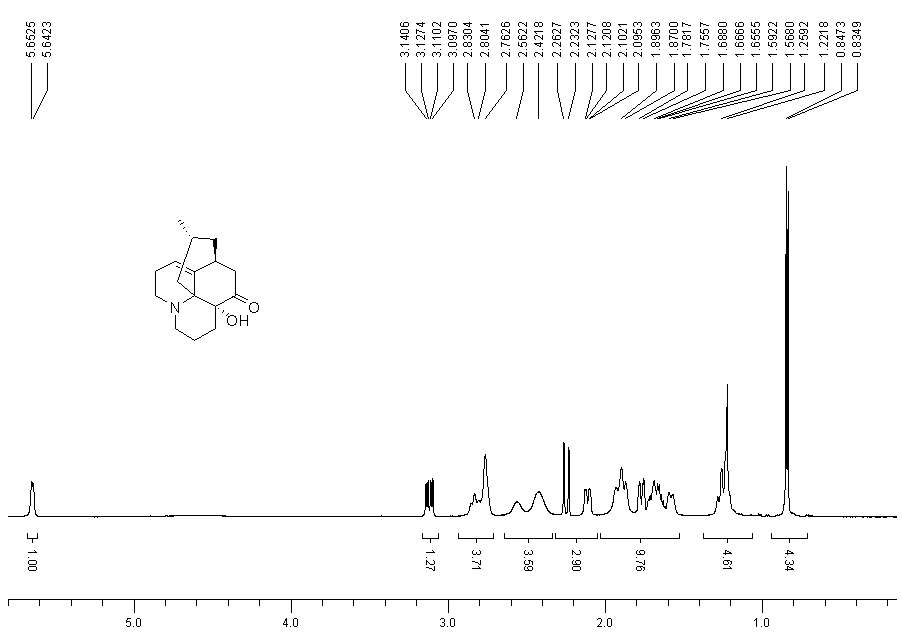


Figure S2. ^13^C and DEPT of compound **1** in CDCl_3_.


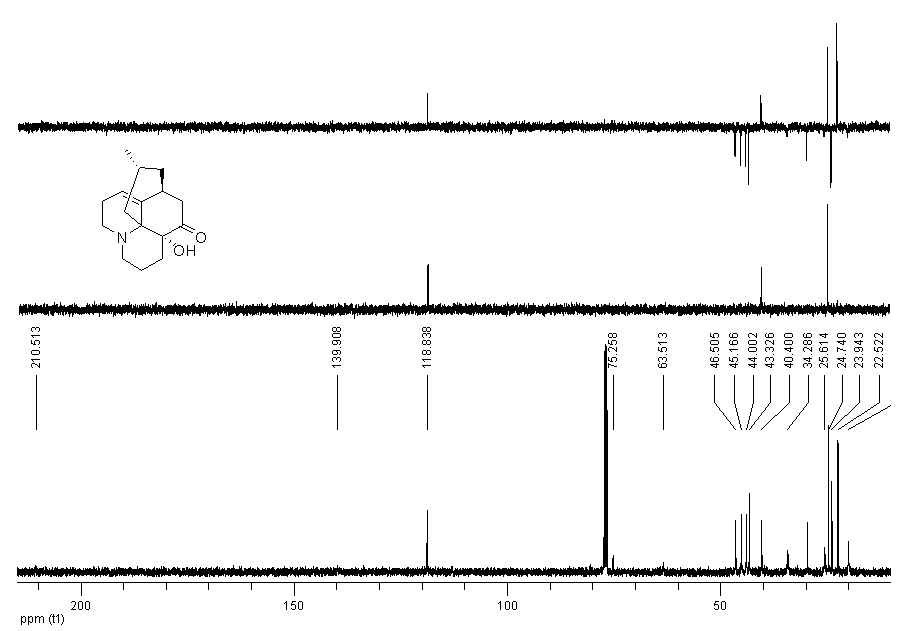


Figure S3. HSQC of compound **1** in CDCl_3_.


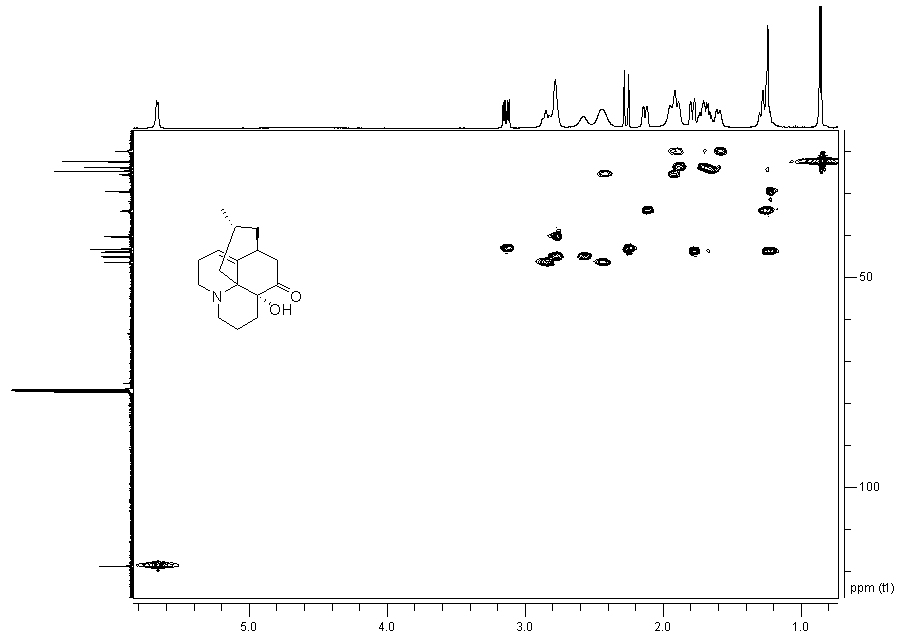


Figure S4. HMBC of compound **1** in CDCl_3_.


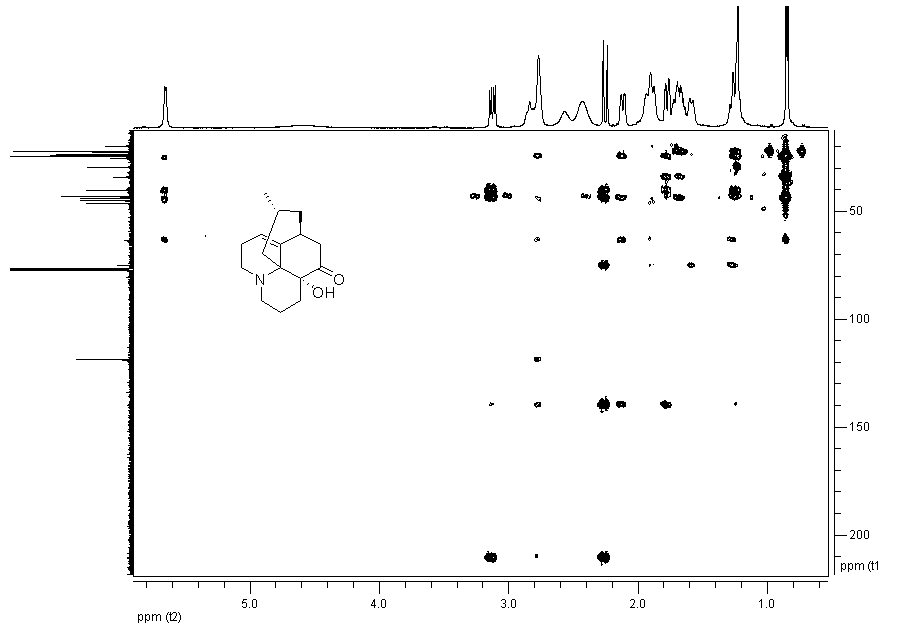


Figure S5. ^1^H−^1^H COSY of compound **1** in CDCl_3_.


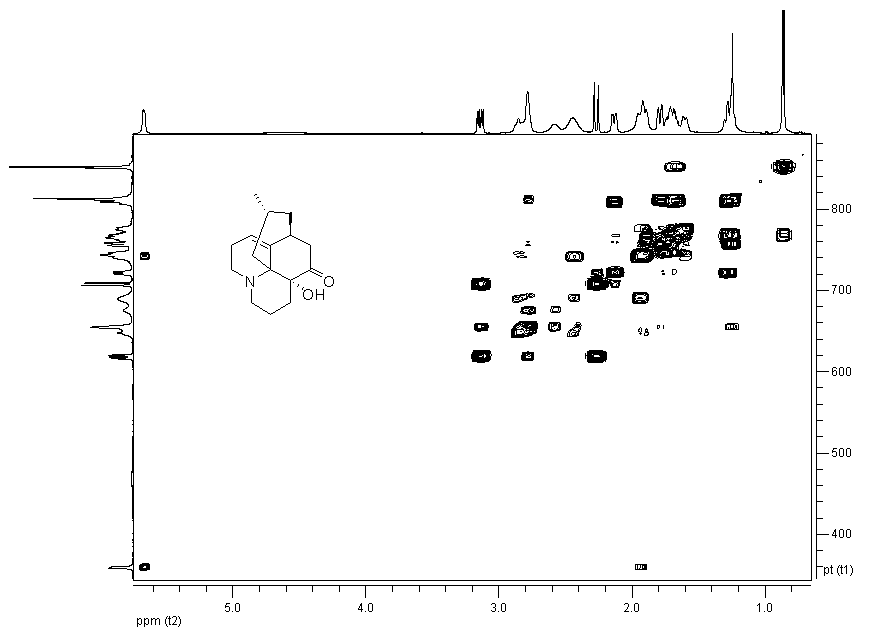


Figure S6. HRESIMS of compound **1** in CDCl_3_.


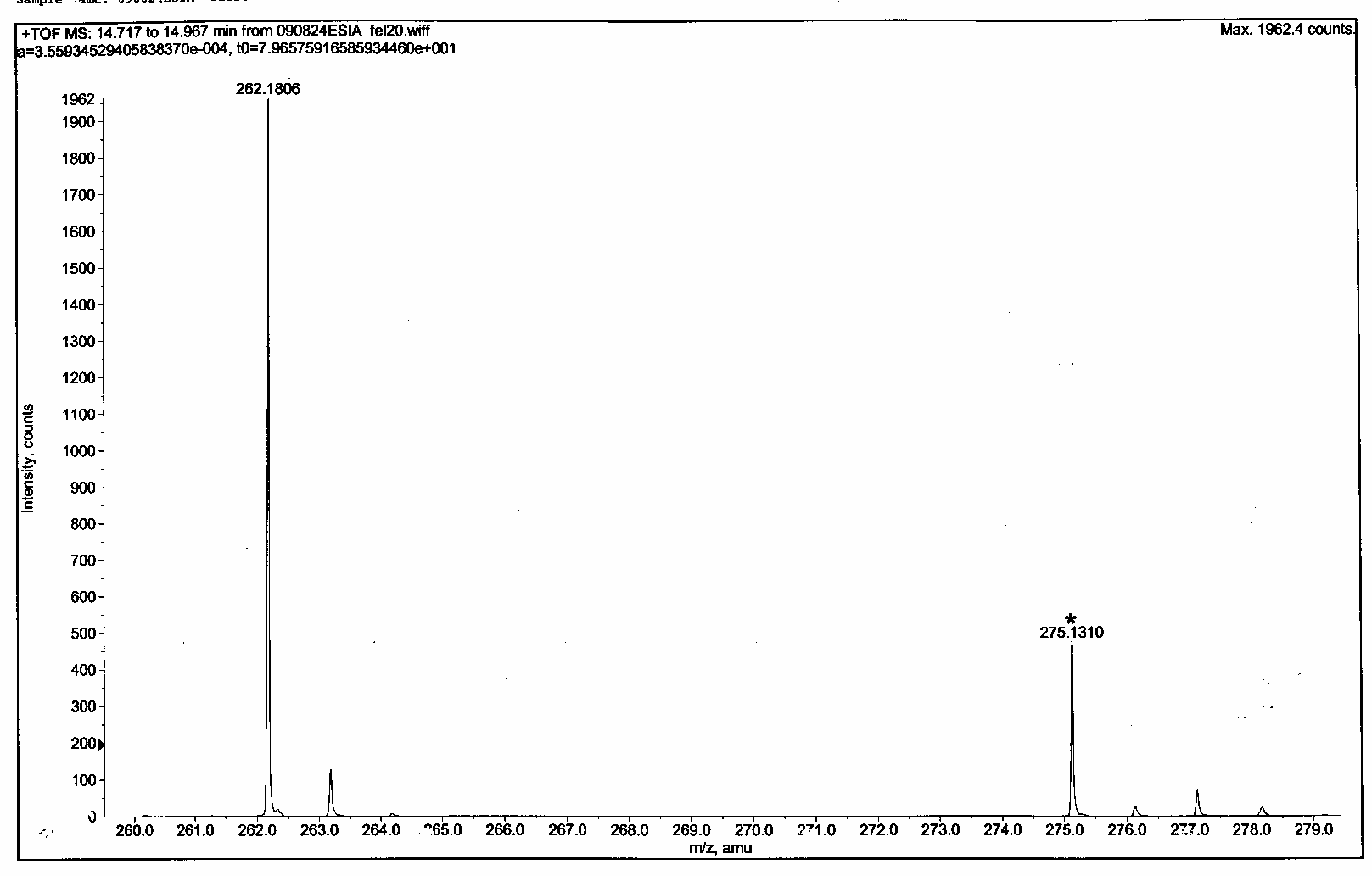


Figure S7. ^1^H of compound **2** in CDCl_3_.


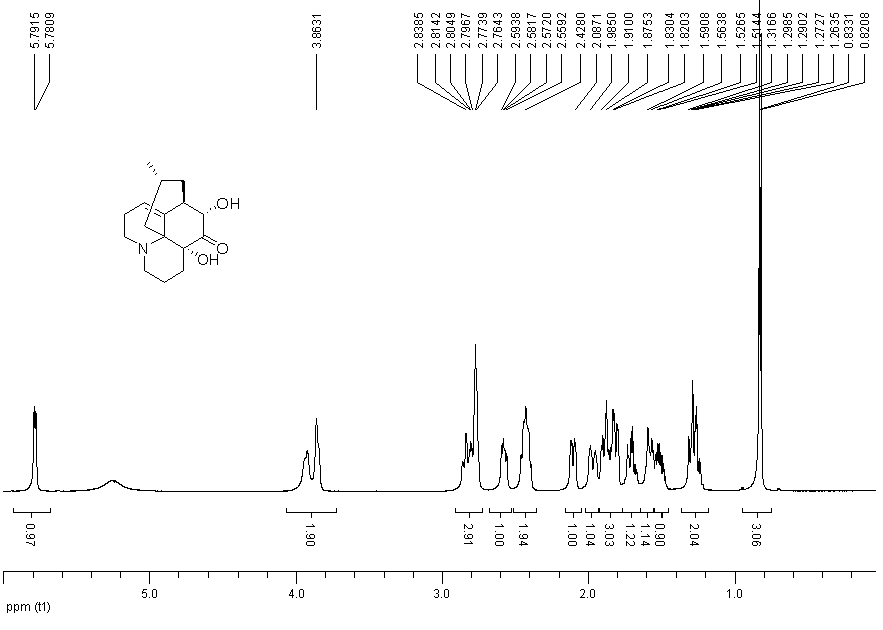


Figure S8. ^13^C and DEPT of compound **2** in CDCl_3_.


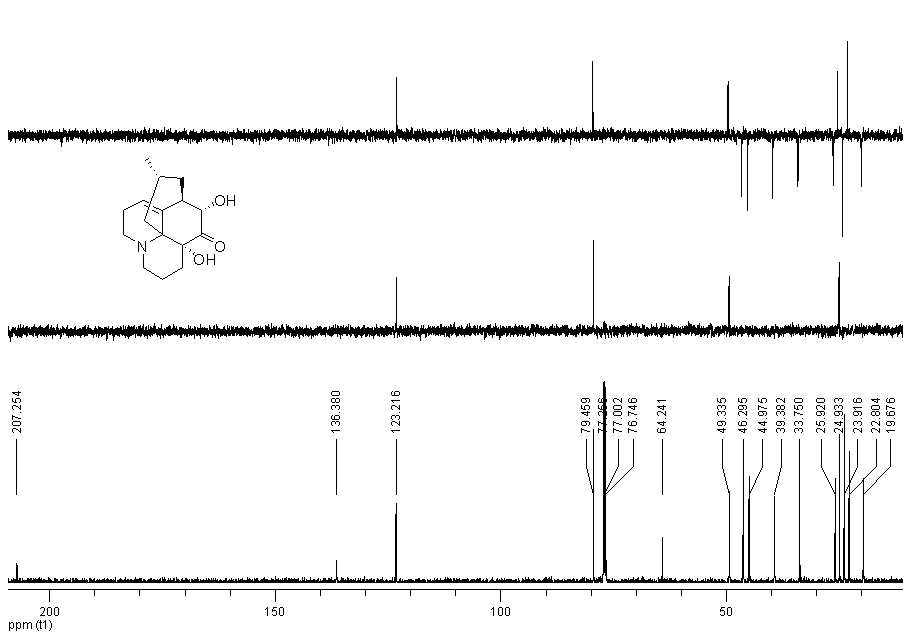


Figure S9. HSQC of compound **2** in CDCl_3_.


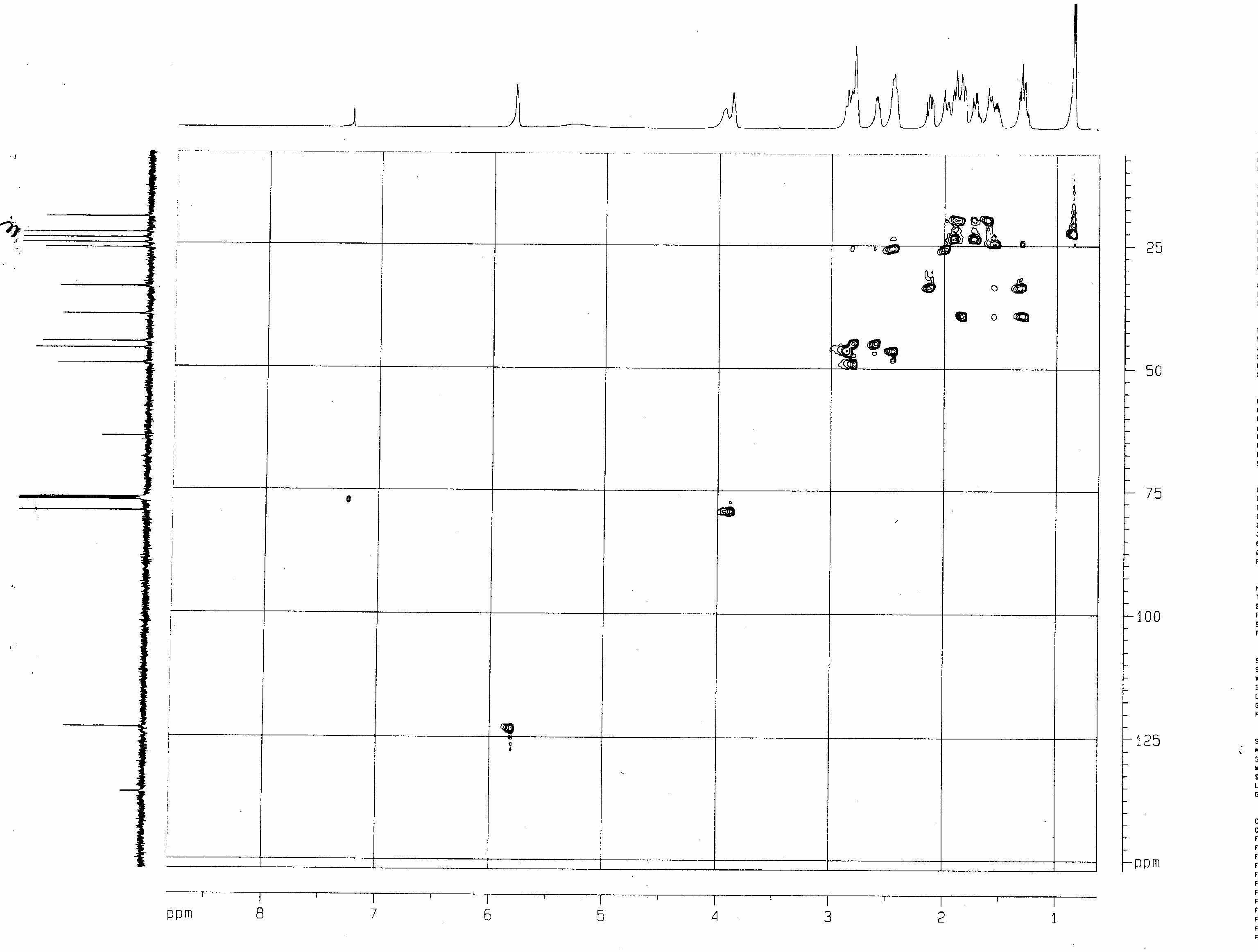


Figure S10. HMBC of compound **2** in CDCl_3_.


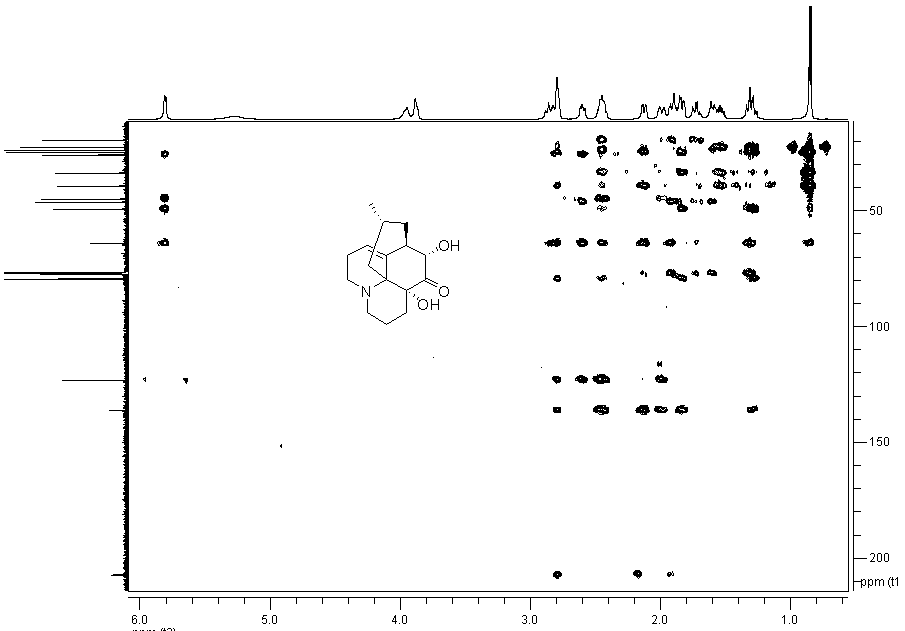


Figure S11. ^1^H−^1^H COSY of compound **2** in CDCl_3_.


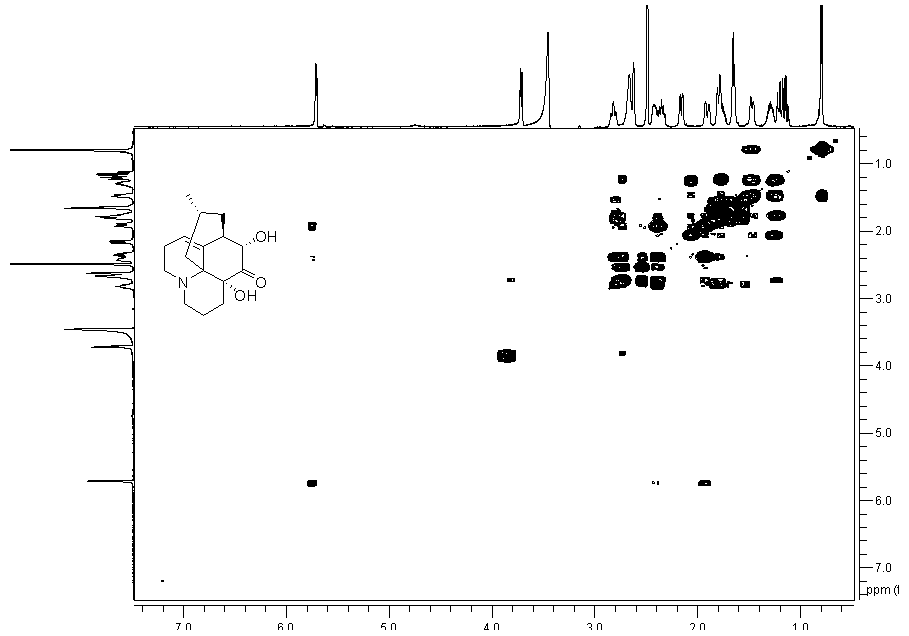


Figure S12. HRESIMS of compound **2** in CDCl_3_.


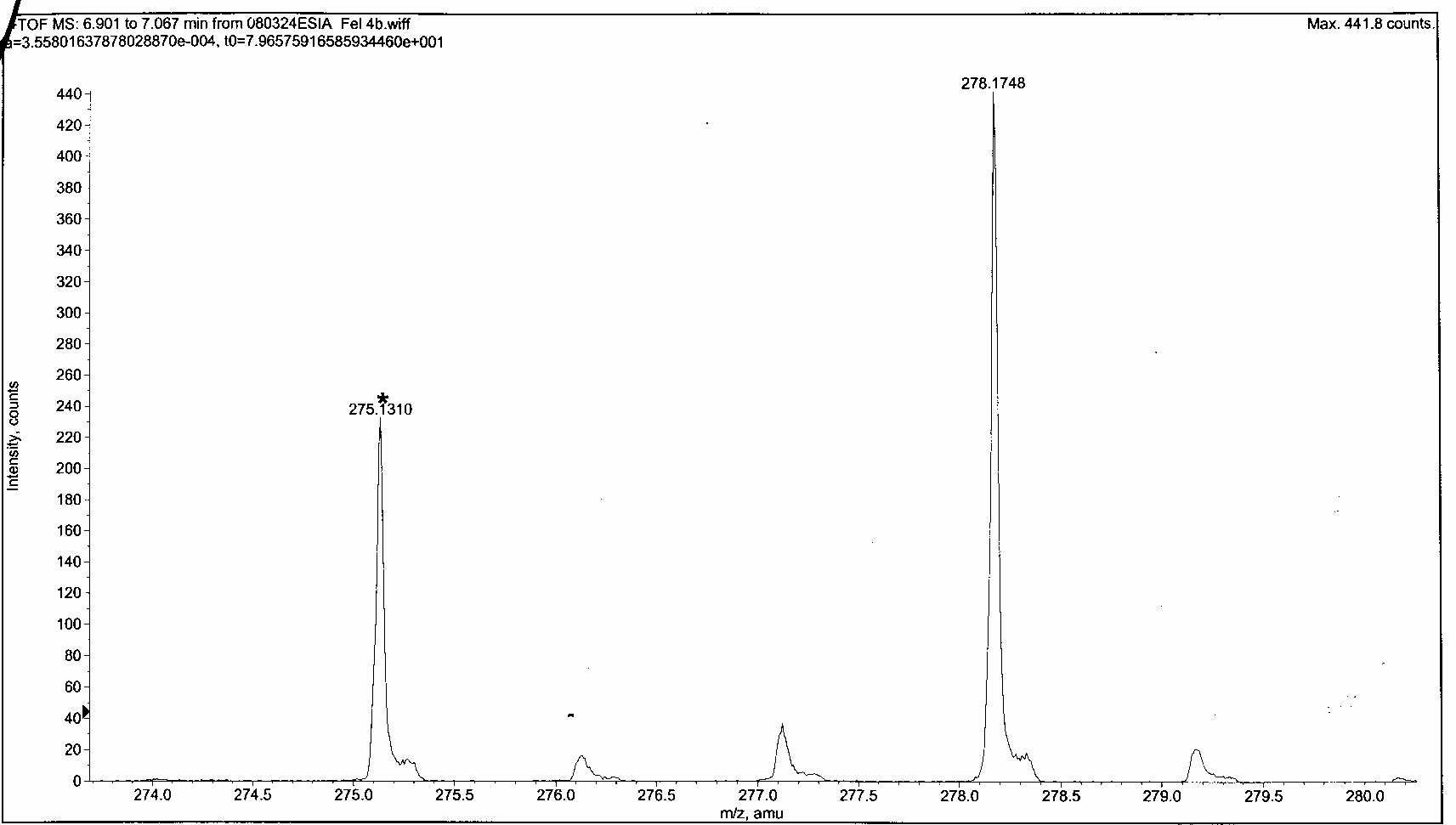


Figure S13. ^1^H of compound **3** in CDCl_3_.


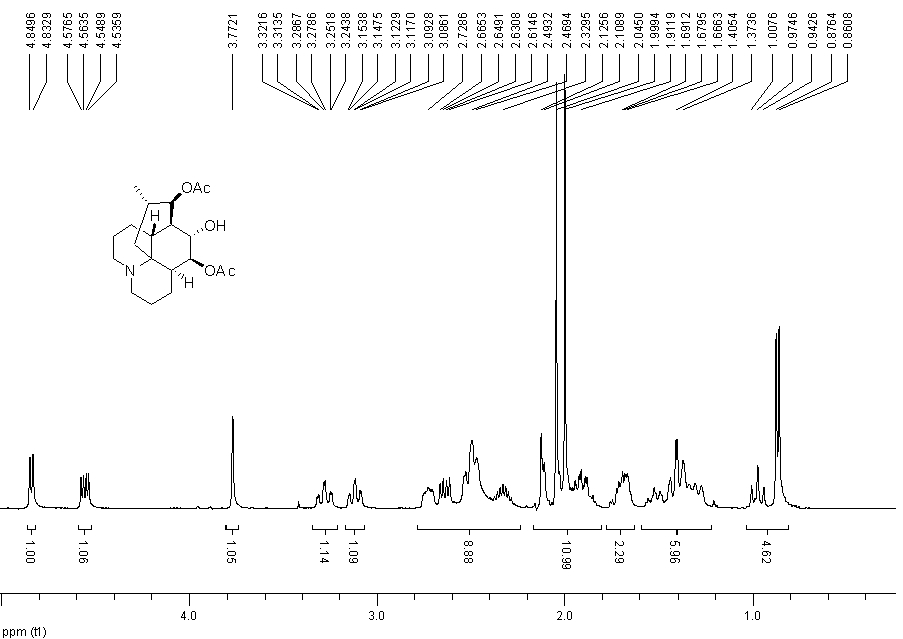


Figure S14. ^13^C and DPTE of compound **3** in CDCl_3_.


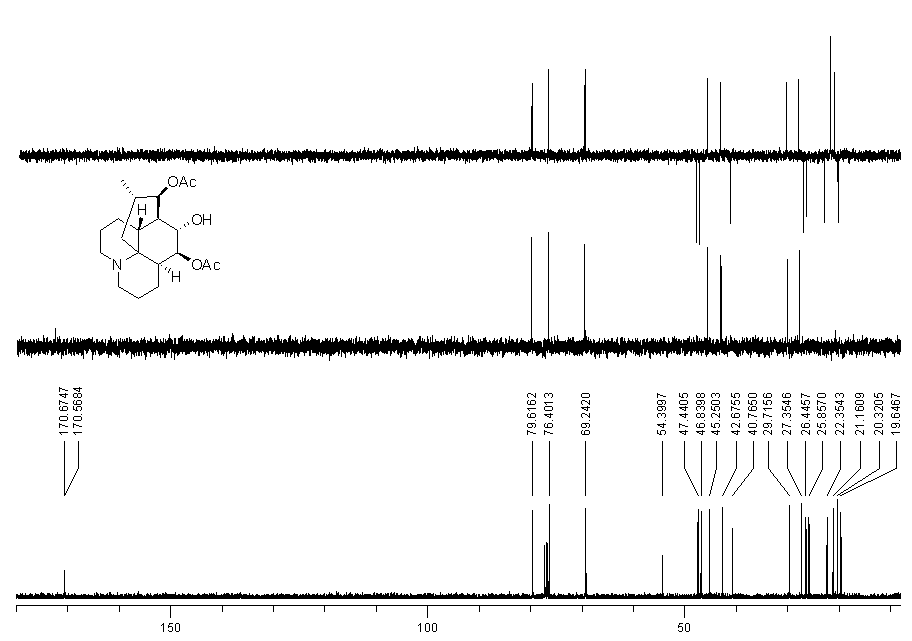


Figure S15. HSQC of compound **3** in CDCl_3_.


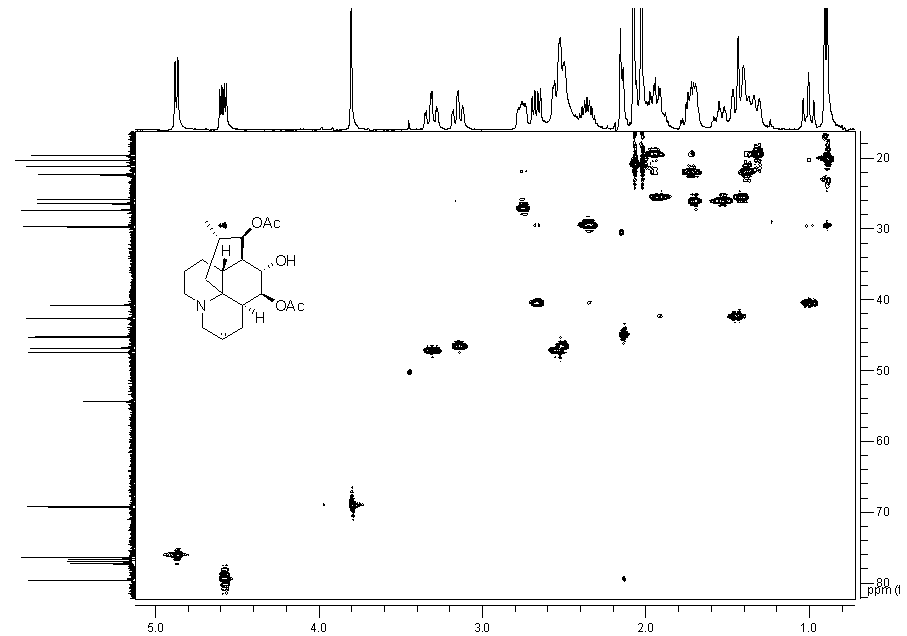


Figure S16. HMBC of compound **3** in CDCl_3_.


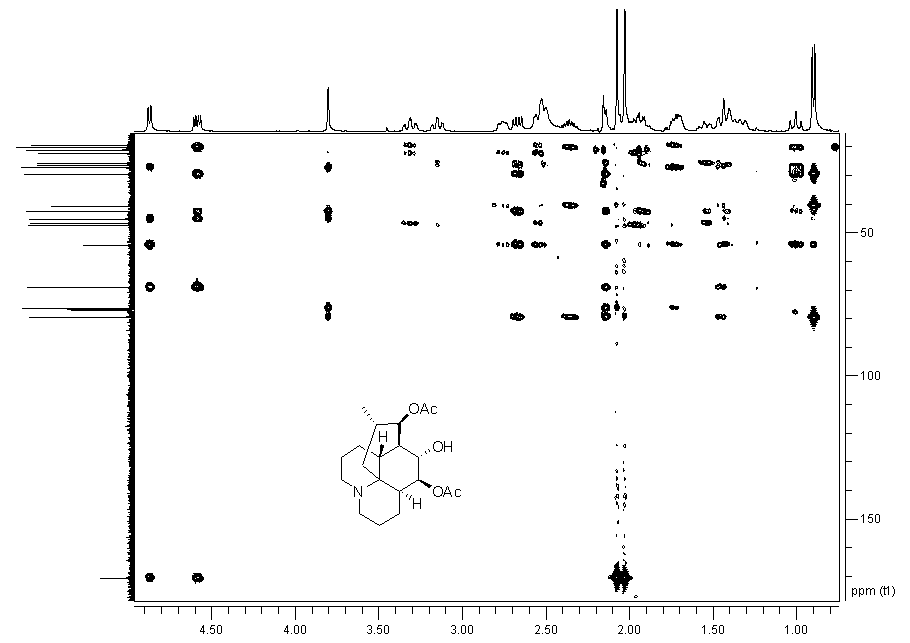


Figure S17. ^1^H−^1^H COSY of compound **3** in CDCl_3_.


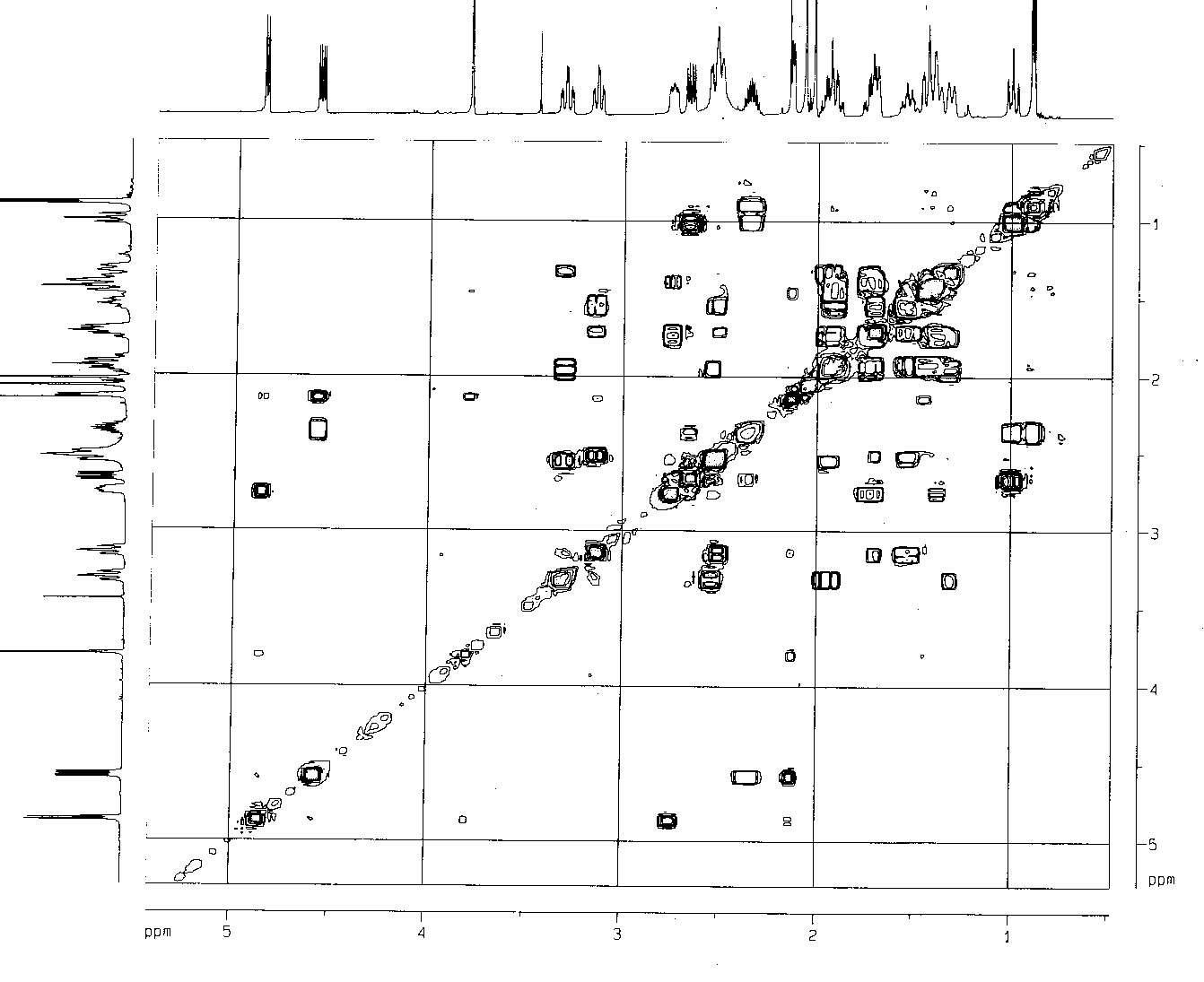


Figure S18. HRESIMS of compound **3** in CDCl_3_.


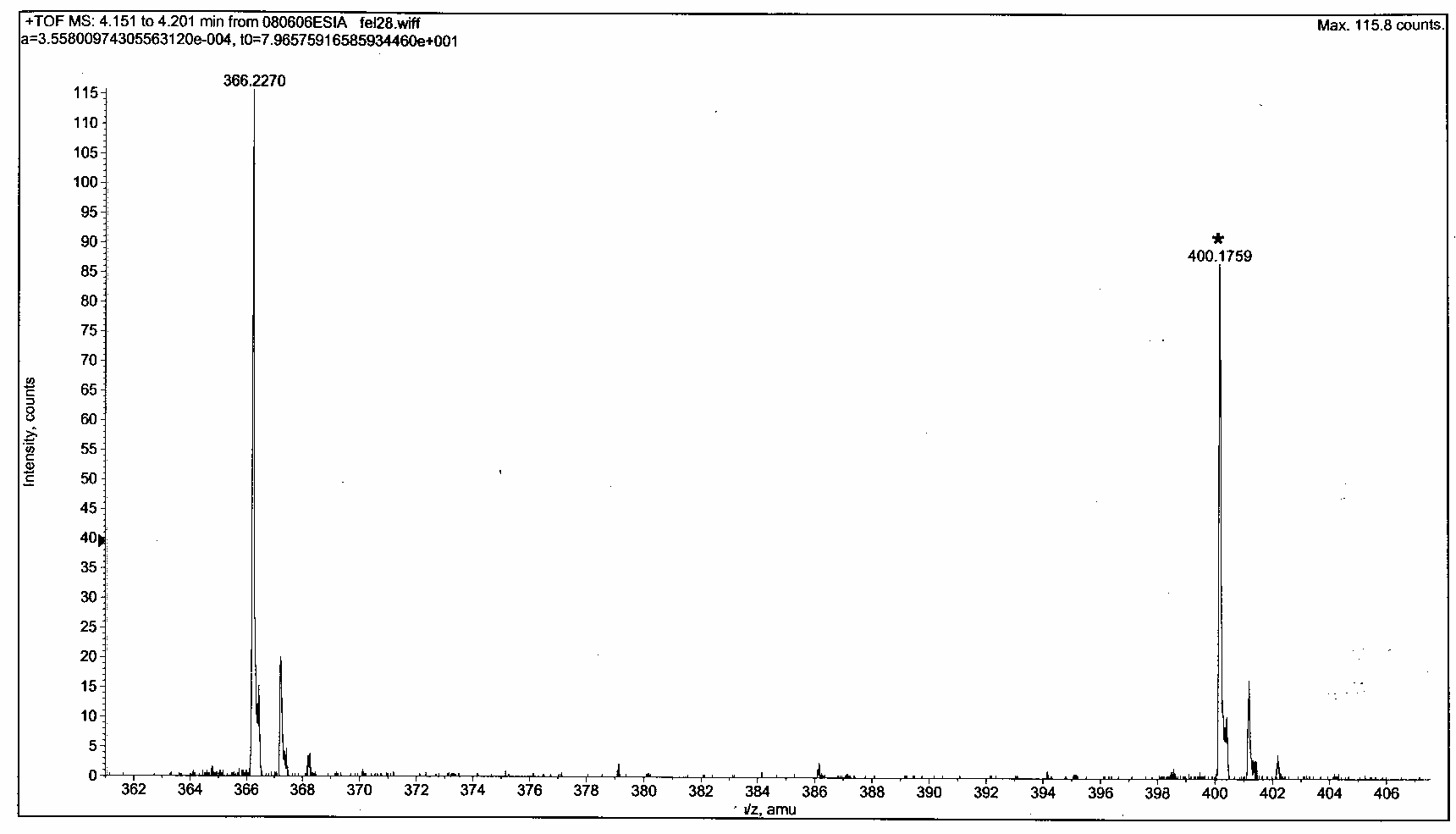


Figure S19. ^1^H NMR of compound **4**.


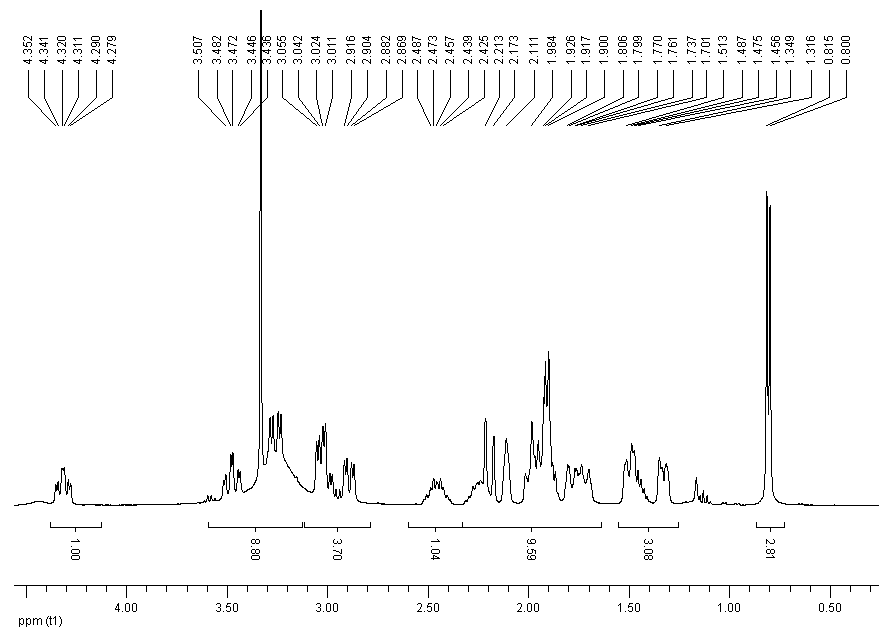


Figure S20. ^13^C and DEPT NMR of compound **4**.


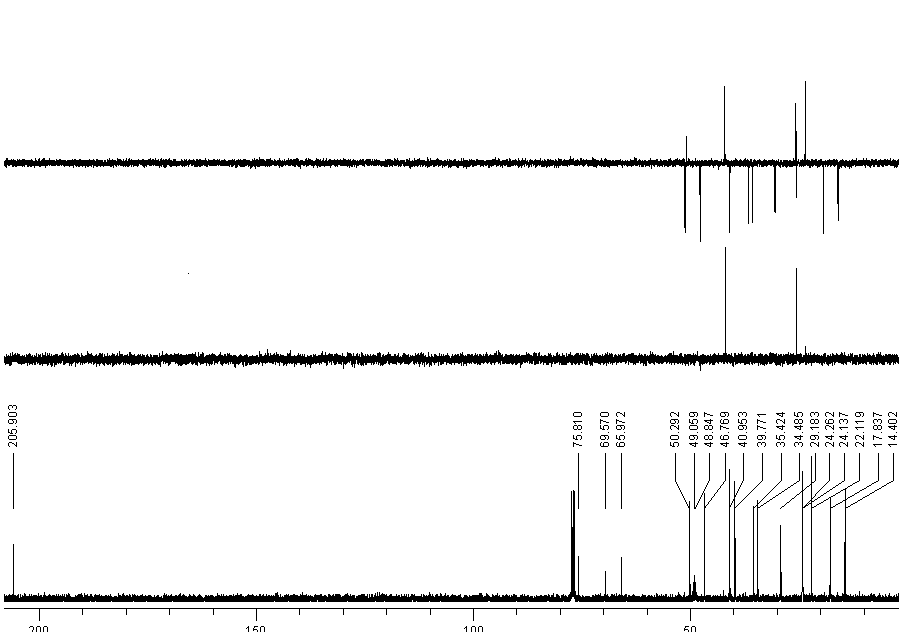


Figure S21. HSQC spectrum of compound **4**.


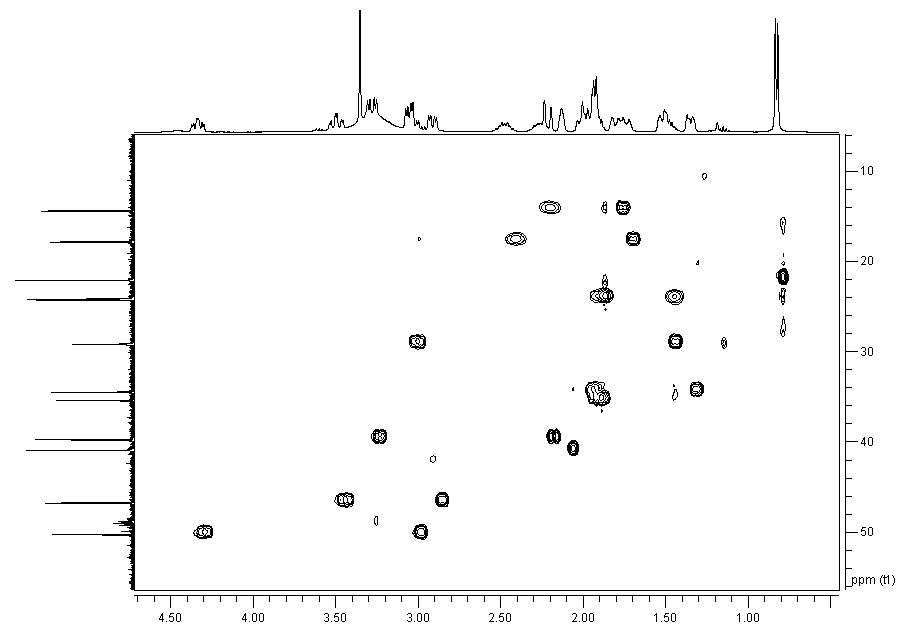


Figure S22. ^1^H-^1^H COSY spectrum of compound **4**.


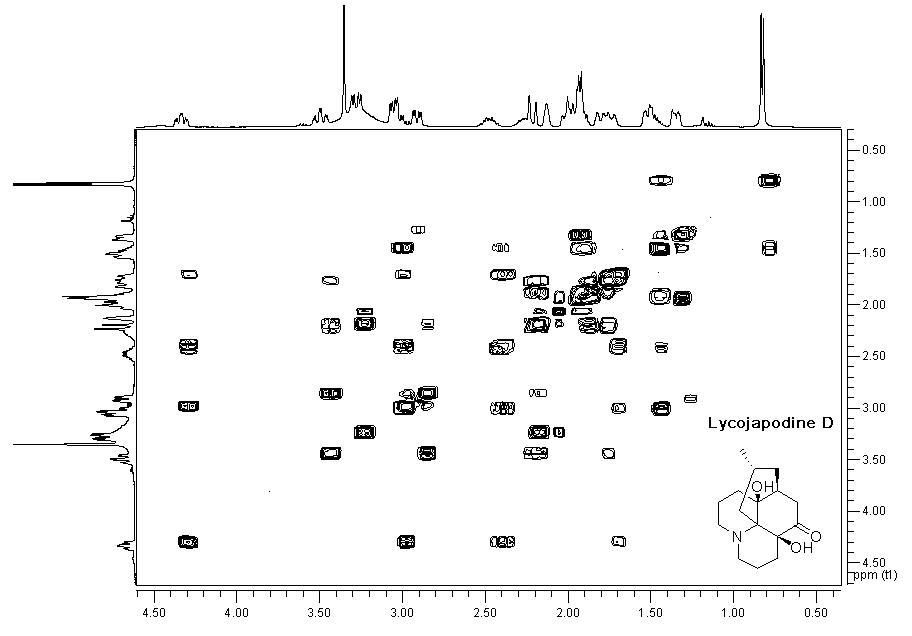


Figure S23. HMBC spectrum of compound **4**.


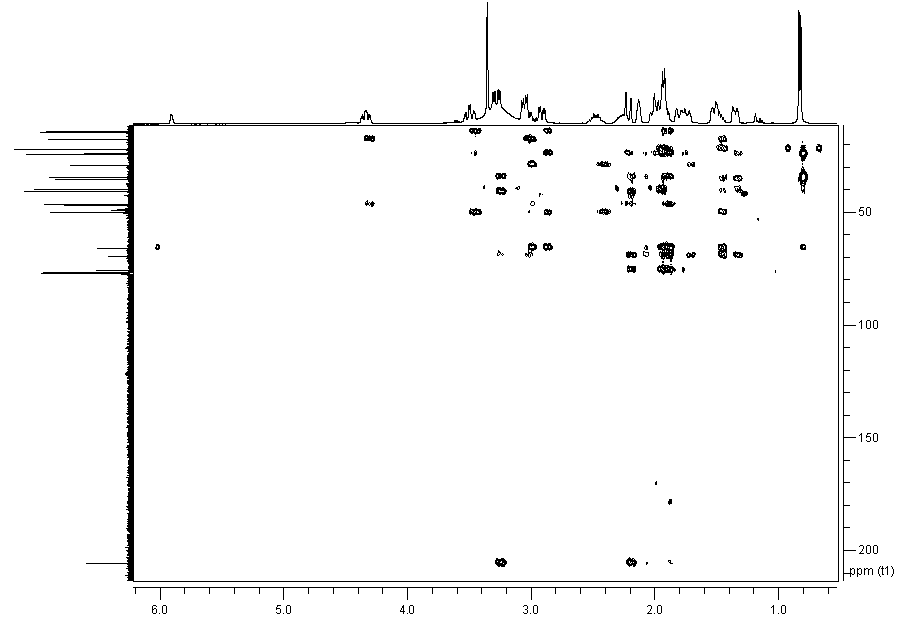


Figure S24. ROESY spectrum of compound **4**.


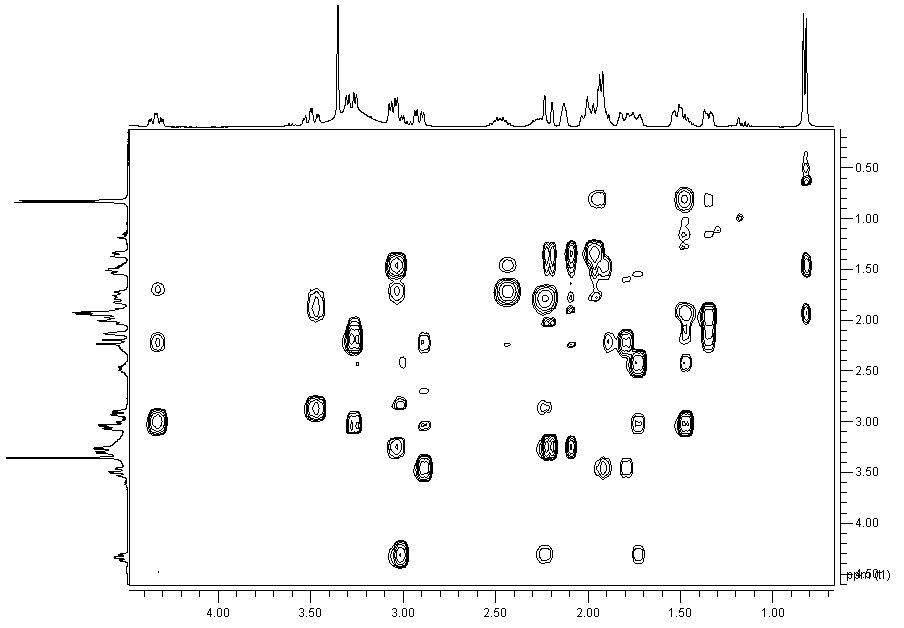


Figure S25. HRESIMS spectrum of compound **4**.


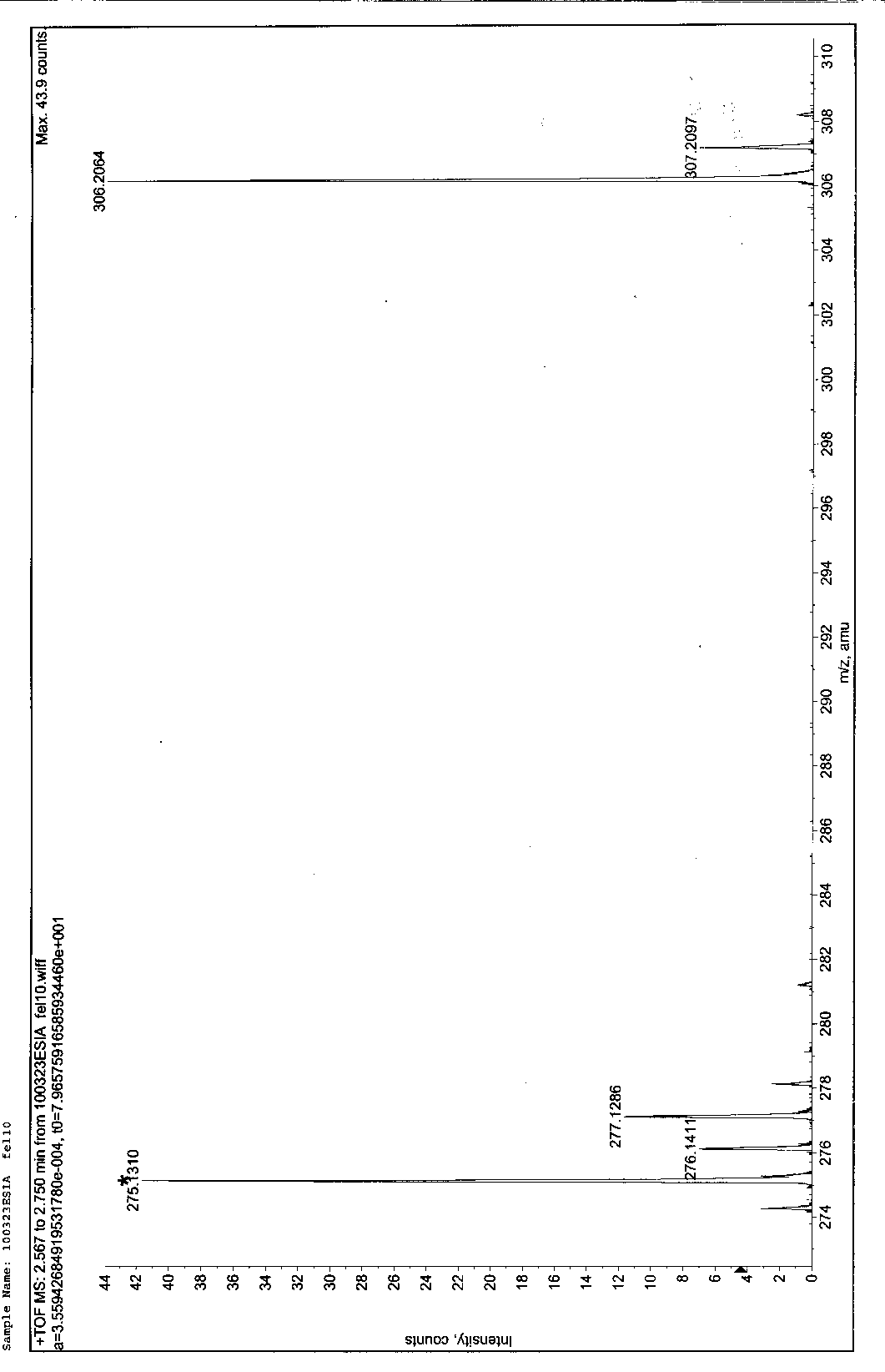

Supplement: Supplementary file 1 — Supplementary material 1 (DOC 716 kb) [file 13659_2014_27_MOESM1_ESM.doc]
